# The Developing Microbiome From Birth to 3 Years: The Gut-Brain Axis and Neurodevelopmental Outcomes

**DOI:** 10.3389/fped.2022.815885

**Published:** 2022-03-07

**Authors:** Hannah E. Laue, Modupe O. Coker, Juliette C. Madan

**Affiliations:** ^1^Department of Epidemiology, Geisel School of Medicine at Dartmouth, Hanover, NH, United States; ^2^Rutgers School of Dental Medicine, The State University of New Jersey, Newark, NJ, United States; ^3^Department of Pediatrics and Psychiatry, Children’s Hospital at Dartmouth, Lebanon, NH, United States

**Keywords:** microbiome, neurodevelopment, autism, neurobehavior, development

## Abstract

The volume and breadth of research on the role of the microbiome in neurodevelopmental and neuropsychiatric disorders has expanded greatly over the last decade, opening doors to new models of mechanisms of the gut-brain axis and therapeutic interventions to reduce the burden of these outcomes. Studies have highlighted the window of birth to 3 years as an especially sensitive window when interventions may be the most effective. Harnessing the powerful gut-brain axis during this critical developmental window clarifies important investigations into the microbe-human connection and the developing brain, affording opportunities to prevent rather than treat neurodevelopmental disorders and neuropsychiatric illness. In this review, we present an overview of the developing intestinal microbiome in the critical window of birth to age 3; and its prospective relationship with neurodevelopment, with particular emphasis on immunological mechanisms. Next, the role of the microbiome in neurobehavioral outcomes (such as autism, anxiety, and attention-deficit hyperactivity disorder) as well as cognitive development are described. In these sections, we highlight the importance of pairing mechanistic studies in murine models with large scale epidemiological studies that aim to clarify the typical health promoting microbiome in early life across varied populations in comparison to dysbiosis. The microbiome is an important focus in human studies because it is so readily alterable with simple interventions, and we briefly outline what is known about microbiome targeted interventions in neurodevelopmental outcomes. More novel examinations of known environmental chemicals that adversely impact neurodevelopmental outcomes and the potential role of the microbiome as a mediator or modifier are discussed. Finally, we look to the future and emphasize the need for additional research to identify populations that are sensitive to alterations in their gut microbiome and clarify how interventions might correct and optimize neurodevelopmental outcomes.

## Introduction

**FIGURE 1:**
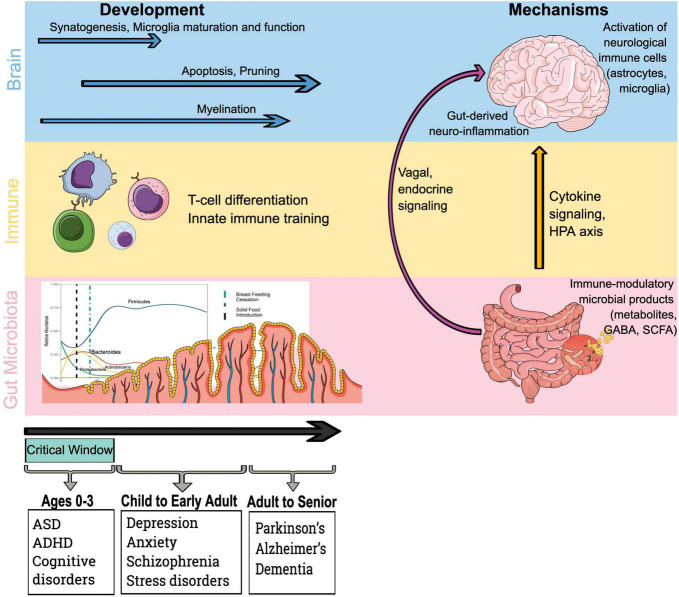
Microbiota, immune and brain development drive mechanistic pathways by which the gut microbiota influences the brain. Existing evidence indicates that early life represents a critical phase in brain development and immune training *via* microbial colonization and early life exposures. Furthermore, immune-mediated mechanisms by which bacteria access the brain and influence neurological outcomes include the impact of immune-modulatory bacterial products on brain. HPA axis, hypothalamic–pituitary–adrenal axis; GABA, γ-aminobutyric acid; and SCFA, short-chain fatty acid.

Infants begin acquiring their intestinal microbiome in earnest at the time of delivery, ultimately achieving an adult-like state by the age of 12–36 months (1–3). It is during this critical window of 0–36 months in neurodevelopment when microbe-human interaction is most variable and parallels the developing immune system (4–7). Interestingly, there is evidence that the gut-brain axis intersects with microbe-immune training mechanisms (Figure 1), as neuroimmune cells communicate with gut microbes and their metabolites during brain development (8, 9). The theory that health across the lifespan is shaped during early sensitive windows, known as the developmental origins of health and disease, is a focus of pediatric molecular epidemiology, and the microbiome likely plays a crucial role in neurodevelopment (Figure 1) (10). During this early fundamental window of 0–3 years – when external environmental influences likely have the most impact on the developing microbiome, and mode of delivery, antibiotics, maternal and neonatal diet can all alter the trajectory of early life microbiome development – the foundation for lifelong health is laid (11–13).

The gut microbiome and its interrelationship with the brain begins *in utero* (14), impacted by maternal medications, stress, vaccinations, chemical exposures, diet, and perinatal exposures such as delivery mode, and perinatal medications (15) and continues throughout the lifespan with the most marked developmental changes in the first years of life. The factors that influence gut microbiome development are thought to impact signaling along the microbiome-gut-brain axis, which has been implicated in a variety of neurological outcomes late in life including neurodegenerative diseases such as Parkinson’s disease and Alzheimer’s (16–18), neuropsychiatric illness such as anxiety and depression (19, 20), and neurodevelopmental disorders such as Autism Spectrum Disorder (ASD) and schizophrenia (21, 22).

Molecular epidemiology studies have begun the important task of evaluating the developmental trajectory of the infant and toddler microbiome in large scale birth cohort studies in an effort to establish norms in healthy populations (12, 23) and investigate the relationship between the microbiome and health outcomes, including neurodevelopment. Although the microbiome includes viruses and fungi, much of the research has focused on bacteria. Throughout this review, we use the word “microbiome” to refer to studies of bacteria. In this review, we summarize work focused on examining the relationship between the microbiome and neurodevelopment.

## Foundations of the Infant Gut Microbiome

The infant microbiome is first impacted by the maternal gestational environment, both in health and in disease; maternal infection during gestation has been implicated in large scale epidemiological studies in subsequent neurodevelopmental disabilities in offspring (such as ASD and schizophrenia) and there is significant reason to hypothesize that the maternal microbiome and metabolome play a role in fetal neurodevelopment and subsequent impairments (24–28). Additionally, the maternal microbiome impacts the neonatal microbiome with clear relationships between maternal gestational diet, perinatal antibiotics, and delivery mode (2, 29–32). Following primary colonization, infant stool microbial taxonomic diversity increases rapidly over the first weeks and months of life, and is influenced by many factors including delivery mode, antibiotics, breast milk, and environmental exposures, leading to a more stable microbiome that has many similarities to family members by age 1–2 years (11). Sex differences have emerged in many investigations of the microbiome-neurodevelopment link, highlighting the importance of research into differential impacts of the microbiome and microbial metabolites on the developing brain (33, 34). Germ-free murine models have played an important role in demonstrating the seminal role gut microbes play in neurodevelopment, both structurally and behaviorally (4, 20, 35–42). Promising animal models targeting development-associated microbes and metabolites offer an exciting perspective on potential novel therapies in humans in the window when brain development is most significant (43). The critical task of establishing norms in the developing microbiome by employing multi-omics investigations (metagenomics, metabolomics) will enable the field to ultimately shift its focus toward understanding dysbiosis vs. health-promoting patterns of microbiome development, identifying opportunities for interventions to treat and prevent disease and optimize neurodevelopmental outcomes.

## Microbiome and Neurodevelopment: The Gut Brain Axis in the Critical Window of Immune and Brain Development

The connection between the gut and the brain, and the potential for bacteria harbored in the gut to influence this connection, has been described in detail elsewhere (44). Most of the related research has focused on adults and with associations clarified in animal models, leaving an important gap: understanding the microbiome-brain connection in the developing brain of early childhood (45). Germ-free murine models have clarified the seminal role gut microbes play in neurodevelopment (36, 39, 46, 47). In the absence of typical colonization of the intestines in early development, murine models demonstrate significant immune deficiencies and severe disorders of brain development and behavioral disturbances, with deficits in learning and memory (4, 37, 41). Germ-free animals have substantial anatomic and physiological differences including underdeveloped hippocampi, cortices, striata and cerebella, defects in microglia, and elevated norepinephrine and serotonin turnover and decreased dopamine turnover in the striatum (20, 35, 36, 38, 40, 42, 48).

Signals from the intestinal microbiome traffic to the central nervous system (CNS) *via* several mechanisms (Figure 1): direct activation of the vagus nerve from the enteric nervous system to the CNS; production of, or induction of, various metabolites that pass through the intestinal barrier and into the circulatory system, where they may cross the blood-brain barrier (BBB) to regulate neurological function; microbial associated molecular patterns (MAMPs, such as lipopolysaccharide or LPS), and metabolites produced by the microbiome can signal to the immune system (49–51). Bacterial commensals in the gastrointestinal tract are also responsible for the production of gamma-aminobutyric acid (GABA), dopamine, noradrenaline, and histamine, all of which are critical in neurodevelopmental processes and neurophysiology (52). The links between the intestine, its microbiome, and the CNS occur *via* the hypothalamic–pituitary–adrenal axis (HPA axis), the enteric and autonomic nervous systems, and hormone signaling – all of which are impacted by microbial metabolites often mediated *via* the systemic immune system (53). The HPA axis, in particular, has been implicated in the gut-brain axis and its relationship to anxiety and stress disorders (52).

Immune cells (and their cytokines) can influence neurophysiology, particularly microglia and astrocytes (54). Microglia protect the brain through cytokine production, phagocytosis, and by activating immune response at all phases of the lifespan (55). During early development, microglia drive synaptic transmission and pruning as well as neuronal circuit development, all of which are impacted by microbial metabolites as described in animal models (56) and may exhibit sex- and age- dependent sensitivity to microbiome signaling (57). Similar to microglia, astrocytes, which are integral to homeostasis of CNS functions (maintenance of the BBB, modulating nutrient transmission), are impacted by microbial metabolites (55, 56, 58).

The role the gut microbiome and its metabolites play in early neurodevelopmental processes is beginning to be elucidated (Figure 1). While much of the work in the gut-brain axis has focused on neurodegenerative processes, neurogenesis is likely impacted by microbes (59). Hippocampal development (responsible for memory formation) is impacted by the intestinal microbiome and its metabolites in germ-free murine models (60), and can be corrected with probiotics in a murine model (61). Neuroinflammation that occurs during early neurodevelopmental windows is associated with upregulation of tumor necrosis factor alpha (TNF-α) expression and, in murine models, impaired memory formation. The work from our group is focused on the role of the gut microbiome in the developmental window from 0 to 3 years when rapid development is occurring, laying the foundation for optimal developmental outcomes (2, 31, 32, 34, 62). We are particularly excited about the study of the gut-brain axis in this critical window because the microbiome is more dynamic, and therefore more likely alterable.

## Role of Inflammatory Signaling Pathways in Neuroinflammation and Neurodevelopment

Systemic inflammation and neuroinflammation are impacted by the intestinal microbiome, particularly in the context of microbial dysbiosis (63, 64). Intestinal inflammation can be the result of dysbiosis and impaired gut epithelial barrier function, resulting in increased transfer of microbial metabolites (65). Metabolites that are derived from gut microbes can impact systemic inflammation *via* stimulation of pro-inflammatory cytokines, resulting in increased permeability of the BBB and neuroinflammation (66). The role of gut-derived neuroinflammation in the context of the developing brain has implications for cognitive development, neurobehavioral and neurodevelopmental disorders [such as ASD and attention-deficit hyperactivity disorder [ADHD)], and neuropsychiatric illness such as anxiety and depression. Clarifying the role of a pro-inflammatory microbiome and its implications for neurodevelopment is critical for investigations aimed at optimizing health outcomes.

The intestinal microbiome modulates neuroimmunity, impacting MAMPs derived from the intestinal microbiome that can drive various aspects of immune function in the periphery (Figure 1). Cytokine signals, such as TNF-α, interleukin 1β (IL-1β), and interleukin 6 (IL-6) can cross the BBB and trigger their production by microglia (54). Interaction of these cytokines with neurons influences physiology and is associated with symptoms of depression (67).

Neuroinflammation is known to affect both brain activity and mental health at all stages of childhood development and in adulthood (45, 68). Inflammation and stress response likely play an important role in major depressive disorder (MDD) and anxiety (20, 69). Studies demonstrate abnormal behavior is related to pro-inflammatory cytokines in depressive patients (70), recovery from which can be enhanced by treatment with anti-inflammatory drugs (71). Furthermore, high levels of pro-inflammatory cytokines can predict future depressive episodes (72). Systemic inflammation has been linked to neurodegenerative and neuropsychiatric conditions such as Alzheimer’s disease and anxiety and depression (45, 68). Microglia are resident immune cells of the CNS that participate in neuroinflammation and are shown to respond to changes in gut microbiome composition (57, 73). Activated microglia impact neural toxicity by releasing reactive nitrogen and oxygen species that damage brain epithelial cells and compromise the BBB. Synthesis of neurotransmitters is hindered during neuroinflammation due to cytokines diverting enzyme cofactors (74). Further work in observational human studies in early life as well as mechanistic investigations in murine models will be critical to clarify the role of neuroinflammation stemming from gut microbes signaling in both typical and atypical neurodevelopment. In the following sections, we discuss our work in the context of others that examine the relationship between the microbiome and specific neurodevelopmental outcomes.

## Microbiome and Social Behaviors That Relate to Autism Spectrum Disorder

One neurodevelopmental outcome that has been a particular focus in microbiome research is ASD. Characterized by impaired social communication and repetitive behaviors in early childhood, ASD is often accompanied by gastrointestinal issues with evidence of immune dysregulation (75, 76). Common comorbid gastrointestinal symptoms such as diarrhea, constipation, and abdominal pain often correlate with the severity of behavioral differences in ASD and are associated with a dysbiosis of the microbiome (77). Interestingly, the causes of ASD remain poorly understood, and are likely the result of a complex interplay of genetic and environmental factors (78–82). The microbiome is an interface between external and internal factors, with the gut acting as a barrier organ. Select genetic and environmental risk factors for ASD could directly cause changes in the indigenous microbiome. Alternatively, the microbiome could be indirectly influenced by other medical co-morbidities associated with ASD, including gastrointestinal issues and immune dysfunction, making it particularly challenging to clarify the direction of association in older children and adults where reverse causation is possible. In particular, dysbiosis of the intestinal microbiome, in addition to immune and gastrointestinal symptoms seen in ASD, can influence neurodevelopment, neural activity and the manifestation of abnormal behaviors characteristic to ASD (21).

### Animal Models

The link between the microbiome and ASD has been investigated in murine and other animal models for nearly a decade (43). Germ-free mouse models often demonstrate that their paucity of microbes associates with ASD-like behaviors such as decreased sociability, particularly in male mice (83), and differential gene expression in the amygdala, the key emotional center mediating responses to social stimuli (84). Of particular interest, some social impairments can be corrected by postnatal colonization of germ-free mice demonstrating the ability to reverse these developmental differences (48). In a seminal translational study by Dr. Sarkis Mazmanian’s team from the California Institute of Technology, transplanted gut microbes from human donors with ASD or neurotypical controls into germ-free mice demonstrated that colonization with ASD microbes induces hallmark ASD behaviors (43). The brains of mice displayed alternative splicing of ASD-relevant genes, and further microbiome and metabolomics evaluation demonstrated that specific taxa and their metabolites, such as phytoestrogens, 5-aminovaleric acid, and taurine, modulate ASD behaviors. After treatment with deficient metabolites, the genetic ASD mouse model showed improved behavior and neuronal excitability in the brain. Their findings support the hypothesis that gut-derived neuroactive metabolites contribute to ASD.

### Epidemiology

Animal models of ASD have been complemented by comparisons of the microbiomes of children with ASD and neurotypical children in clinical populations. Many studies point to an altered gut microbiome in children with ASD, with lower abundances of fermentative bacteria (like *Prevotella*) and lower overall bacterial diversity, leading to the hypothesis that a paucity of beneficial gut microbes impairs neurological health (85, 86). However, the case-control design of these and other studies (87–94) limits our understanding of the direction of the associations between gut bacteria and social behaviors (i.e., reverse causation is possible with ASD-related behaviors altering exposures that change the microbiome). Thus, prospective studies of early life bacterial communities that alter later social behaviors are essential to our understanding of the microbiome-ASD link. In our group’s investigation of early life microbiome and social behavioral outcomes, we studied over 150 subjects from the New Hampshire Birth Cohort Study (NHBCS) over the first 3 years of life, capturing microbiome samples longitudinally and measuring the Social Responsiveness Scale, 2nd edition (SRS-2), which assesses social behavioral deficits and ASD traits on a continuous scale, in 3-year-olds (62). Several keystone bacterial taxa measured at 1, 2, and 3 years associated with SRS 2 scores. Importantly, we also identified metabolic pathways that related to SRS-2 ratings, such as L-ornithine and vitamin B6 biosynthesis. These associations require in-depth investigation into their potential for use as therapeutic interventions, but bacterial byproducts may prove easier to supplement than bacteria themselves.

## Microbiome and Neurobehavioral Outcomes

Similar to ASD, researchers began studying the relationship between the microbiome and other neurobehavioral outcomes such as ADHD, depression, and anxiety after noting that gastrointestinal symptoms including food sensitivities were often comorbid with these outcomes (95, 96). The early-life gut microbiome is a driver of HPA axis programming (97) and contributes to stress responses across the lifespan (98). Previous reviews have highlighted the roles of gut peptides (99), inflammation (100), and microbiome-mediated gut production of neurotransmitters (101) in moderating the role of the microbiome in neurobehavior.

### Animal Models

Germ-free animal models support a connection between the microbiome and neurobehavior (102, 103). Several studies have demonstrated differences in anxiety and/or depression behaviors in germ-free mice (43). Transplantation of stool from donors with MDD into germ-free mice was sufficient to induce depressive behavior compared to those who received transplants from healthy controls (104). Furthermore, disturbances in carbohydrate and amino acid metabolism were noted in the comparison of fecal, serum, and hippocampal metabolomic profiles from depressed and control mice, demonstrating the systemic effects of gut microbiome dysbiosis. Similarly, murine models of anxiety have shown differences in behavior related to microbiomes; specifically, germ-free status is associated with less anxious behavior, but increased corticosterone (19). Animal models of behavior can provide key mechanistic insights, but are ultimately limited by their ability to replicate human phenotypes.

### Epidemiology

Cross sectional studies in children and adults have identified associations between patterns in the gut microbiome and outcomes such as anxiety (105–107), ADHD (108, 109), and depression (110–112). However, many of these are measured at one timepoint later in childhood or adulthood and reverse causation is possible. Furthermore, biologic evidence suggests that early life microbiome development in parallel with the HPA axis may be especially important for anxiety and depressive symptoms, indicating that later stool samples may not reflect relevant microbial differences. To address these gaps, our lab examined the relationships between the infant microbiome at three early life timepoints and continuous measures of internalizing and externalizing symptoms at 3 years of age measured using the Behavioral Assessment System for Children, 2nd edition preschool form (BASC-2), which captures adaptive and maladaptive behaviors in 2–5 year-olds (34). Due to differences in neurodevelopment between males and females, we explored whether the associations between the microbiome and behavior differed depending on child’s sex (33). In one of the first studies to investigate the earliest timepoints of microbiome composition in a longitudinal study we identified relationships between the microbiome at three early life time points (6 weeks, 1 year, 2 years) and anxiety, depression, social, and hyperactivity symptoms. While prior studies support our findings, they do not include microbiome measurements in infancy, making ours unique in highlighting an opportunity for meaningful intervention in the first few weeks and months of life.

## Microbiome and Cognitive Development

From birth to age 3 years, 86 billion neurons are developing and 100 trillion connections are being made between neurons. During this critical window of brain development, neural circuits are sensitive to environmental inputs. Social/emotional and cognitive development occurs in parallel, requiring regulation from molecular signaling pathways. Basic neurodevelopmental processes are modulated by the gut microbiome as demonstrated in colonization of germ-free animals or depletion of gut bacteria by antibiotics in animal models. Specifically, the processes that are modulated include BBB formation and integrity, neurogenesis, microglia maturation, myelination, and expression of neurotrophins, neurotransmitters, and their respective receptors (43, 113).

### Epidemiology

There are very few studies in humans investigating the gut microbiome in the first months and years of life in relationship to cognitive development. In a recent study of infant microbiome patterns in association with cognitive development, one of the first of its kind, specific patterns in the gut microbiome at 1 year were associated with cognitive development at 2 years of life (114). A *Bacteroides*-dominant microbiome in the first year of life was associated with receptive/expressive language, and overall higher diversity was associated with lower scores, with associated structural differences noted on brain MRI at 2 years. In this study, volumes of specific brain regions (left precentral gyrus, left amygdala, right angular gyrus) at 2 years associated with alpha diversity. The study highlighted no associations with overall volume, gray matter, white matter, cerebral spinal fluid (CSF), ventricle volumes or other regional volumes (114). Two other studies have examined the microbiome in relation to scales that measure cognitive development. In the Vitamin D Antenatal Asthma Reduction Trial (VDAART) study the Ages and Stages Questionnaire was employed to assess motor and problem-solving skills at 3 years in relation to 3- to 6-month-old microbiomes (6). Toddlers with *Bacteroides*-dominant microbiomes had lower fine motor scores. Interestingly, this adverse effect was primarily observed in infants whose mothers received higher vitamin D supplementation during pregnancy. In a cross-sectional assessment of microbiomes and cognition in Chinese toddlers, mental and psychomotor development, measured with the Bayley Scales of Infant Development, were both associated with a microbiome cluster characterized by higher relative abundances of *Faecalibacterium*, *Sutterella*, and *Clostridium XIVa* (115). These early studies highlight the potential contribution of the microbiome to cognitive development. While less relevant to the pediatric developmental window, there is evidence that the microbiome is also involved in cognitive decline, suggesting the microbiome’s important influence across the life course (116). Additional epidemiologic and mechanistic studies could inform our understanding of the gut-brain connection.

## Environmental Influences on the Developing Microbiome: Gut Bacteria as a Mediator in Environmental Epidemiology

The exposome, defined by the National Institute of Environmental Health and National Institute for Occupational Safety and Health as the measure of all of an individual’s exposures over the life course, is an important concept when we think about microbiome research and how it fits into an epidemiological framework. Common environmental toxicants are implicated in disrupting the typical developmental trajectory of the microbiome in early life (117, 118) and are also associated with adverse neurodevelopmental health outcomes (119); the microbiome is likely a mediator or moderator between environmental exposures and health outcomes. Lead, arsenic, manganese and other environmental toxicants have been implicated in neurodevelopmental disorders in young children and we and others have demonstrated that these exposures also influence the microbiome (118, 120), including that harmful and nutrient exposures can act antagonistically on the same bacteria (121). This has fascinating implications for the prevention of neurodevelopmental disorders and highlights opportunities to leverage alteration of the early-life microbiome to optimize developmental outcomes in young children.

## Future Directions: The Gut-Brain Axis Is Alterable and in This Critical Window Has Lifelong Implications for Optimizing Health Outcomes

### Susceptible Populations

As highlighted by our findings with the BASC-2 (34), the health effects related to changes in the gut microbiome are not consistent across all populations, and consideration of host characteristics such as sex is important in identifying individuals who are most at risk or would benefit most from microbiome interventions. Further, periods when hormone levels are in flux (e.g., puberty, pregnancy, or postnatally) may indicate windows of neuro-susceptibility to microbial dysbiosis (122–127). In addition to hormonal windows of susceptibility, infants are a particularly vulnerable population among whom cognitive or behavioral changes resulting from microbial dysbiosis will have the most impact across the lifespan. The effects of genetic polymorphisms that make individuals susceptible to environmental exposures (e.g., AS3MT) may be ameliorated by bacteria that can supplement host metabolism of toxins. Investigation of microbial dysbiosis and stressful life events could identify synergies between these dual stressors.

### Interventions

One example of targeting the gut microbiome as a potential focus to improve both gastrointestinal and neuropsychiatric/behavioral symptoms is in ASD. Antibiotics have been studied, with initially very exciting benefits of oral vancomycin (which is not absorbed but impacts the microbiome significantly) for both gastrointestinal symptoms and behavior (128). Sadly, the benefits were lost just weeks after antibiotics were discontinued. Promising results have emerged from pilot investigation of fecal microbiome transplants (FMT) in children and adolescents with ASD, which demonstrated improvement in both gastrointestinal symptoms as well as neurobehavioral symptoms, effects that persisted at least 8 weeks after treatment ended. The results demonstrated that the average GI distress score decreased by 82%, and ASD-related behaviors improved during and after the study, including the Childhood Autism Rating Scale decreasing by 22% during and 24% 8 weeks post treatment. At the same time, the Vineland Adaptive Behavior Scales (VABS-II), a measure of adaptive skills such as communication, socialization and daily living skills resulted in an average developmental age increase of 1.4 years (129). Ongoing larger studies are likely to provide important information about optimal treatment timing and duration (129). Novel applications of FMT in neuropsychiatric and neurodegenerative diseases have been explored in murine models, including Alzheimer’s and Parkinson’s disease, schizophrenia and depression in addition to neurodevelopmental disorders such as ASD (103). Novel investigations have begun to target neuropsychiatric disorders (130), with some promising results.

In murine models, augmenting the gut microbiome with *Bacteroides fragilis* alone alters the gut microbiome and microbial metabolites, resulting in decreased gut permeability and improved ASD-associated behaviors (131). Use of probiotics in children with ASD have demonstrated somewhat mixed results, with somewhat unclear mechanisms (132). Probiotics in general have been shown to improve gut integrity, alter serum microbial-derived metabolites, and have anti-inflammatory properties (133). Likewise, psychobiotics (probiotics targeting neuropsychiatric and psychological conditions) have shown promise in the treatment of mental health disorders by stimulating the production of gut-derived neurotransmitters, short chain fatty acids (such as butyrate, propionate) and gut derived hormones (134). Early work has identified potential opportunities for targeted probiotic supplementation (primarily *Lactobacillus* and *Bifidobacterium* strains) in the perinatal period, during pregnancy, in preterm infants, and in term infants at high risk for the development of immune-mediated disease, with important evaluations of safety and efficacy of maternal transfer to offspring with subsequent implications for neurodevelopment (135).

## Conclusion

The volume and breadth of research on the role of the microbiome in neurodevelopmental and neuropsychiatric disorders has expanded greatly over the last decade, opening doors to new models of mechanisms of the gut-brain axis and therapeutic interventions to reduce the burden of these outcomes. Studies have highlighted the window of birth to 3 years as an especially sensitive window when interventions may be the most effective. Future research is needed to elucidate mechanisms, identify susceptible populations, and clarify effective treatments for specific neurodevelopmental disorders. Ideally, neurodevelopmental disorders and neuropsychiatric illness could be prevented rather than treated, and harnessing the powerful gut-brain axis during the critical developmental window of 0–3 years offers tremendous opportunities for future investigations into the microbe-human connection and the developing brain.

## Author Contributions

HEL, MOC, and JCM: writing – original draft preparation and writing – review and editing. All authors contributed to the article and approved the submitted version.

## Conflict of Interest

The authors declare that the research was conducted in the absence of any commercial or financial relationships that could be construed as a potential conflict of interest.

## Publisher’s Note

All claims expressed in this article are solely those of the authors and do not necessarily represent those of their affiliated organizations, or those of the publisher, the editors and the reviewers. Any product that may be evaluated in this article, or claim that may be made by its manufacturer, is not guaranteed or endorsed by the publisher.
